# Supplementary material for: An intranasal influenza virus-vectored vaccine prevents SARS-CoV-2 replication in respiratory tissues of mice and hamsters
Source: Nat Commun. 2023 Apr 12;14:2081. doi: 10.1038/s41467-023-37697-1 (PMC10092940; doi:10.1038/s41467-023-37697-1)
Supplement: Supplementary file 1 — Supplementary Information [file 41467_2023_37697_MOESM1_ESM.pdf]

Supplementary Fig.S1

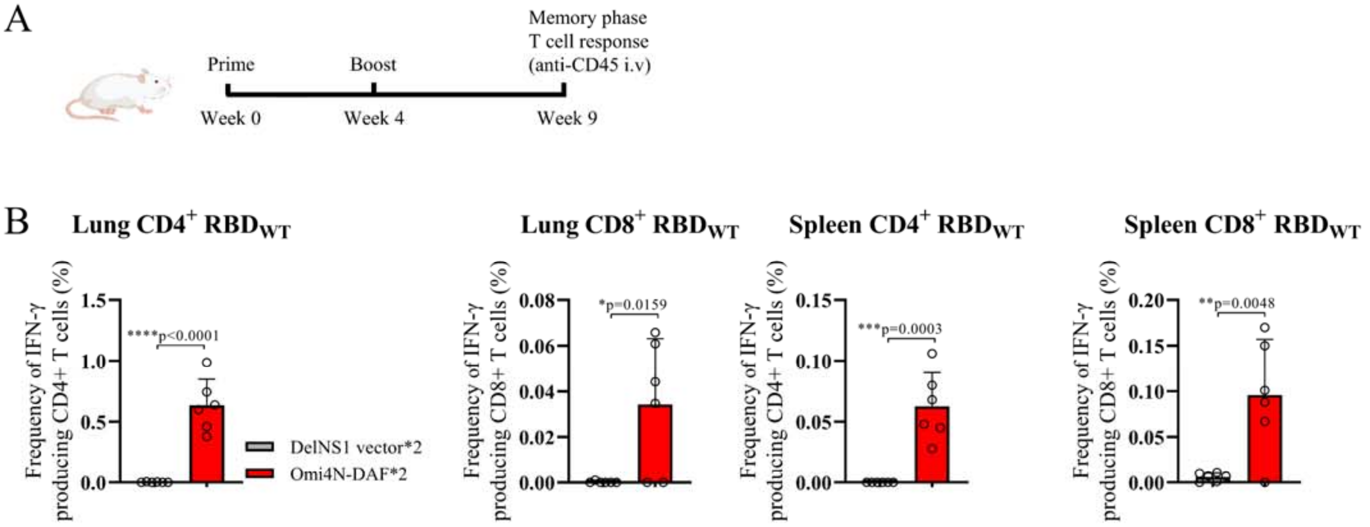

Fig. S1 Memory CD4+ and CD8+ T cell response induced by DelNS1-RBD4N-DAF LAIV.
(A) Schedule for immunization of BALB/c mice. At week 5 after the second immunization, 2 µg of PerCP-Cy5.5 conjugated CD45-specific antibody was injected i.v. via the tail vein 5 min before sacrifice. Lung cells and splenocytes were obtained and stimulated with or without spike peptide pools (Supplementary Table 2) overnight in the presence of BFA. Surface markers (CD69, CD103, CD4, CD8 and Zombie) were stained, and cells then fixed and permeabilized. Intracellular IFNγ was then stained with specific antibodies. (B) Omi4N-DAF induced tissue resident memory T (Trm) cell responses in lungs (CD45- IFN-γ+ CD69+ CD4+ T cells and CD45- IFN-γ+ CD69+ CD103+ CD8+ T cells) and spleens (CD45- IFN-γ+ CD4+ and CD8+ T cells). Percentages of T cell subsets in immunized mice (n = 6 for each group) were compared. Error bars represent mean ± SD. Statistical comparisons between means were performed by Student's t-test (2-tailed): **** $p < 0.0001$, *** $p < 0.001$, ** $p < 0.01$, * $p < 0.05$. Mouse cartoons created with BioRender.com.

Supplementary Fig.S2

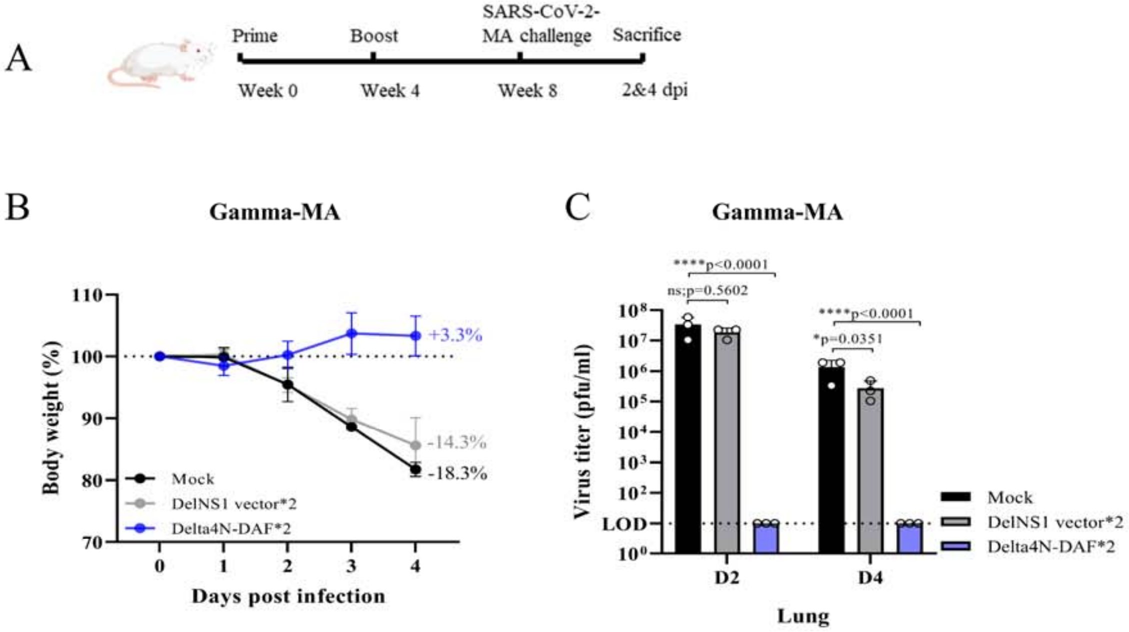

Fig. S2 Protection against challenge with mouse-adapted SARS-CoV-2 Gamma strain (Gamma-MA) in mice prime-boost immunized with DelNS1-RBD4N-DAF vaccine.

(A) Illustration of schedule of immunization and SARS-CoV-2 virus challenge for BALB/c mice. Mice were intranasally prime-boost vaccinated with Delta4N-DAF (2 x 10^6 pfu), DelNS1 vector (2 x 10^6 pfu) or PBS (n=6 for each group) and then challenged with the mouse-adapted SARS-CoV-2 strain Gamma-MA (5 x 10^4 pfu) 4 weeks after boost immunization. (B) Body weight changes over time. (n=6 for each group). (C) Virus titers in the lungs were measured at 2 dpi (n=3 for each group) and 4 dpi (n=6 for each group). LOD: lower limit of detection. Error bars represent mean ± SD. Statistical analysis was performed using one-way ANOVA followed by Dunn's multiple comparisons test: **** $p < 0.0001$, * $p < 0.05$, ns: not significant. Mouse cartoon created with BioRender.com.

Supplementary Fig.S3

A

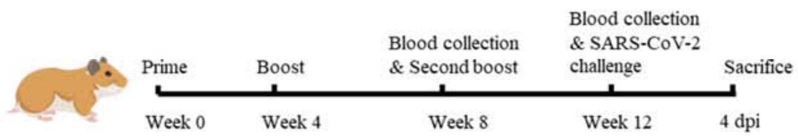

Regimen	Prime	Boost	Second Boost
Mock	/	/	/
BNT*2	BNT162b2(i.m.)	BNT162b2(i.m.)	/
BNT*3	BNT162b2(i.m.)	BNT162b2(i.m.)	BNT162b2(i.m.)
BNT*2+Delta4N	BNT162b2(i.m.)	BNT162b2(i.m.)	CA4-DelNS1-Delta4N-DAF(i.n.)
BNT*2+Omi4N	BNT162b2(i.m.)	BNT162b2(i.m.)	CA4-DelNS1-Omi4N-DAF(i.n.)

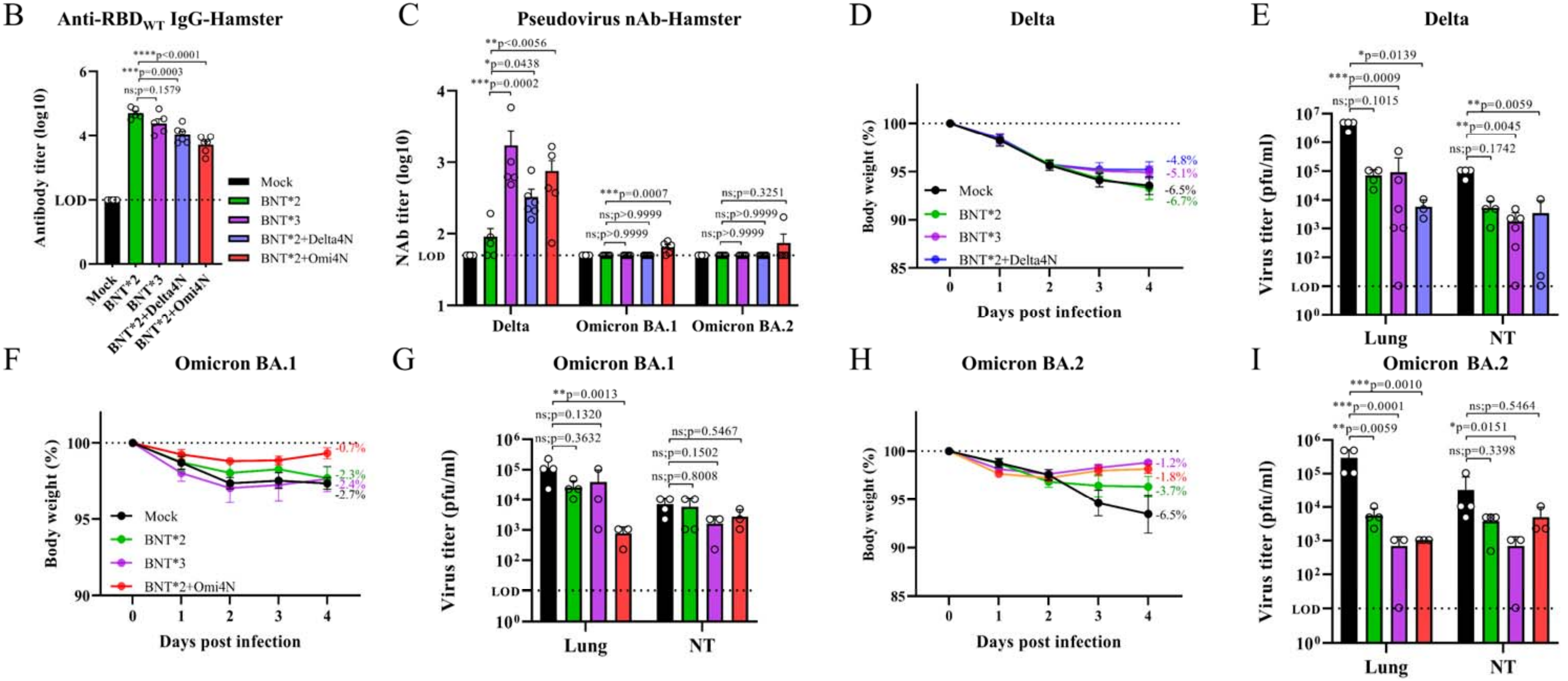

Fig. S3 Protective efficacy of DelNS1-RBD4N-DAF LAIVs when used as "mix and match" boosters following two doses of BNT162b2 mRNA vaccine in hamsters. (A) Hamsters were vaccinated using different regimens: two doses of BNT162b2 mRNA vaccine (BNT*2), three doses of BNT162b2 mRNA vaccine (BNT*3), two doses of BNT162b2 mRNA vaccine with a third dose of the Delta4N-DAF (BNT*2+Delta4N) or two doses of BNT162b2 mRNA vaccine with a third dose of the Omi4N-DAF (BNT*2+Omi4N). Sera samples were collected 14 days after the last immunization and tested for (B) anti-S1 RBD-specific IgG titers (BNT*2 (n=5), BNT*3 (n=5), BNT*2+Delta4N (n=6), BNT*2+ Omi4N (n=6) and mock (n=6)), (C) neutralization titers against pseudotyped viruses displaying Delta, Omicron BA.1 or Omicron BA.2 spike proteins (BNT*2 (n=5), BNT*3 (n=5), BNT*2+Delta4N (n=6), BNT*2+Omi4N (n=5) and mock (n=5)). Hamsters were challenged with Delta, Omicron BA.1 or Omicron BA.2 SARS-CoV-2 variants at 1×10^4 pfu per hamster 4 weeks after their last immunization. (D, F and H) Body weight changes following SARS-CoV-2 virus challenge of hamsters immunized according to the different vaccine regimens. (E, G and I) Virus titers in the lungs and nasal turbinates (NT) of hamsters were measured at 4 dpi. LOD: lower limit of detection. Error bars represent mean $\pm$ SD. Statistical comparisons between means were performed by Student's t-test (2-tailed): **** $p < 0.0001$, *** $p < 0.001$, ** $p < 0.01$, * $p < 0.05$, ns: not significant. Mouse cartoon created with BioRender.com.

Supplementary Fig.S4

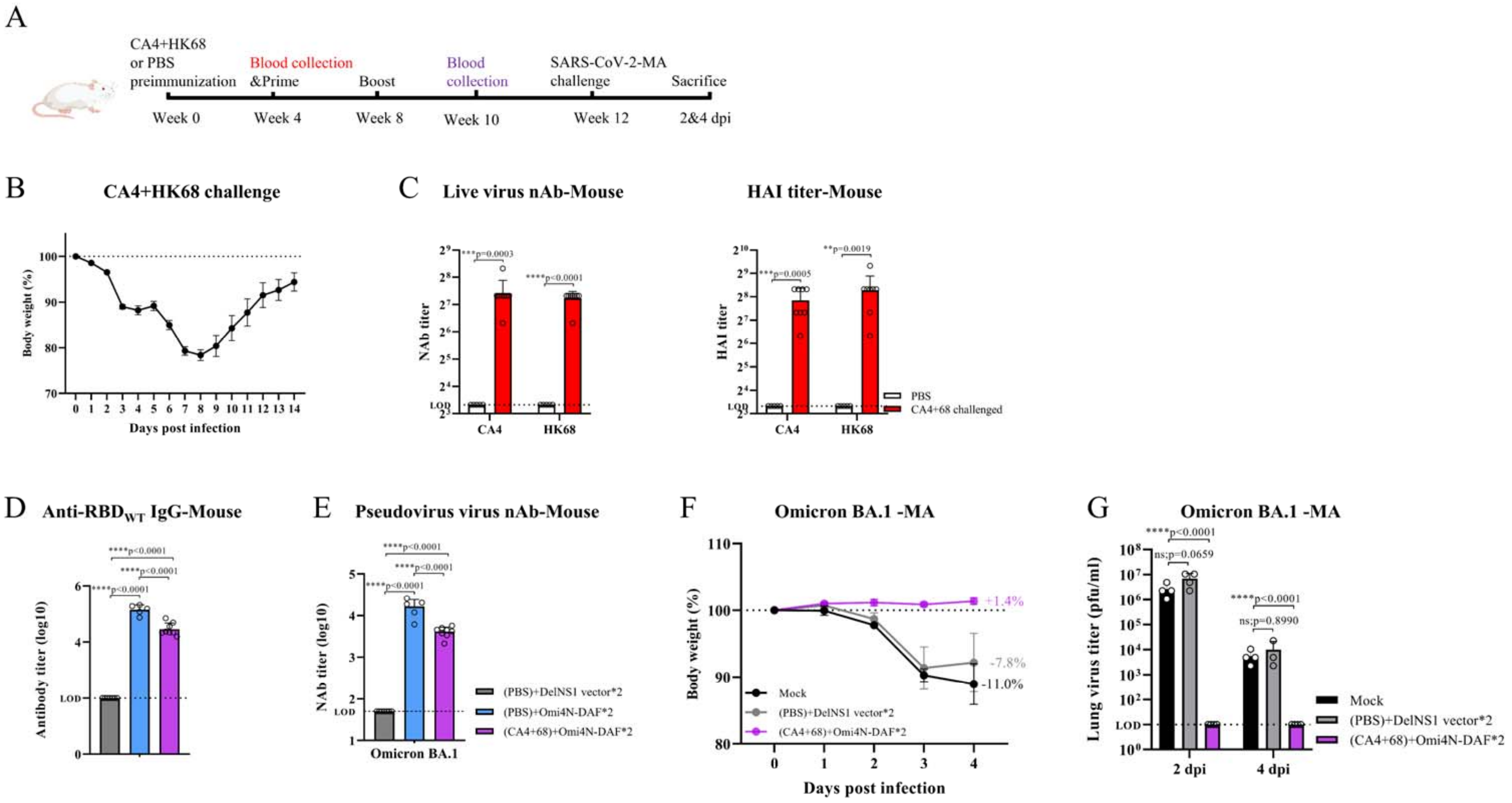

Fig. S4 Effect of pre-existing anti-influenza immunity on the immunogenicity and protective ability of Omicron DelNS1-RBD4N-DAF LAIV.

(A) Schedule for preimmunization, immunization and virus challenge of BALB/c mice. Mice were challenged with a sublethal dose containing both wild type CA4 and HK68 viruses ($2x10^4$ pfu of each virus per mouse) or control PBS (n=8 for each group). (B) Body weight and disease symptoms were monitored for 2 weeks. (C) At week 4 post infection, sera were collected for testing of neutralization titers and hemagglutination inhibition (HAI) titers against live influenza viruses CA4 (H1N1) or HK68 (H3N2). Starting at week 4 after preimmunization, mice were intranasally prime-boost vaccinated 4 weeks apart with 2×10^6 pfu of Omi4N-DAF or DelNS1 vector ((PBS)+DelNS1 vector (n=7), (PBS)+Omi4N-DAF (n=5), (CA4+68)+Omi4N-DAF (n=8)). Sera were collected 14 days after the second immunization for (D) testing of anti-S1 RBD-specific IgG titers, and (E) neutralization titers against pseudotyped viruses displaying Omicron BA.1 spike proteins. The mice were then challenged with Omicron-MA 4 weeks after boost immunization. (F) Body weight and disease symptoms were monitored for 4 days. (G) Virus titers in the lungs were measured at 2 dpi and 4 dpi. LOD: lower limit of detection. Error bars represent mean ± SD. Statistical analysis for (C) was performed by Student's t-test (2-tailed), and statistical analysis for (D), (E) and (G) was performed by one-way ANOVA with Dunn's multiple comparisons test: **** $p < 0.0001$, *** $p < 0.001$, ** $p < 0.01$, ns: not significant. Mouse cartoon created with BioRender.com.

Supplementary Fig.S5

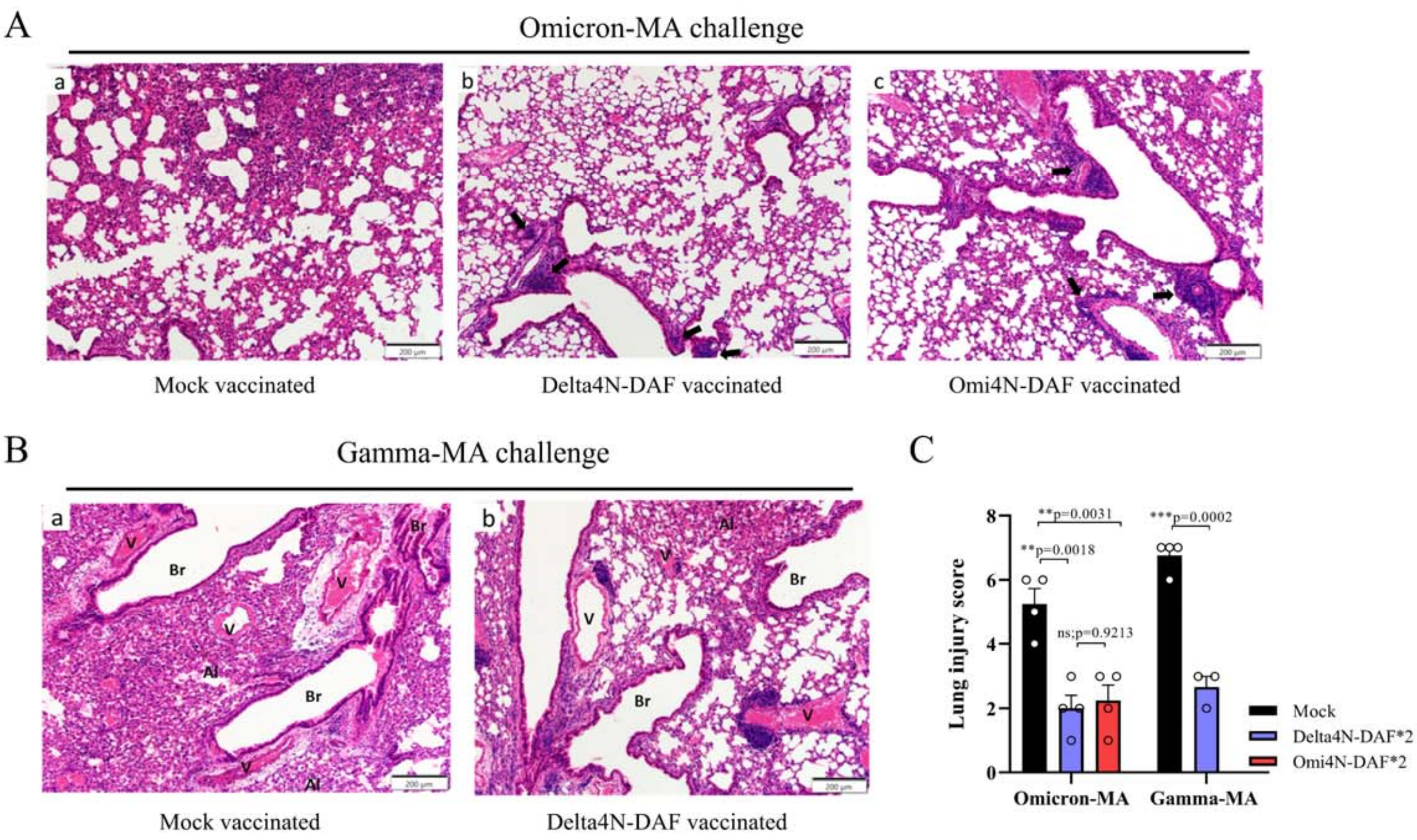

Fig. S5 Histopathological analysis of lung pathology in immunized-challenged mice.
Mice were intranasally prime-boost vaccinated with Delta4N-DAF (2 x 10^6 pfu), Omi4N-DAF (2 x 10^6 pfu) or PBS (Mock) (n=4 for each group). Four weeks after boost immunization, mice were challenged with the mouse-adapted SARS-CoV-2 strains Omicron-MA (1 x 10^5 pfu) or Gamma-MA (5 x 10^4 pfu) (n=4 for each group). Lungs were collected, fixed, processed into paraffin blocks, and sections H&E stained at 4 dpi. (A) Mock-vaccinated or immunized mice challenged with Omicron-MA virus. a. The lung mainly showed alveolar wall thickening due to increased immune cell infiltration and alveolar septum vessel congestion. b. The lung section showed foci of infiltrating immune cells around the bronchioles (arrows); the alveolar structure appeared normal. c. The lung section appeared to have a largely normal alveolar structure. Several foci of immune cells were observed around the blood vessels (arrows). (B) Mock-vaccinated or immunized mice challenged with Gamma-MA virus. a. The lung mainly showed alveolar wall edema and mild alveolar space infiltration with mononucleated cells. Severe to mild perivascular edema (V) and with some immune cell infiltration was also seen. Bronchial epithelium damage and infiltration were mild (Br). b. The lung section showed a patchy area of alveolar infiltration (Al), and several foci of infiltrating immune cells around the blood vessels (V). The bronchioles showed mild infiltration while the epithelium was largely normal (Br). Br, Bronchiole; V, blood vessel, Al, alveolar structure. (C) The lung sections were quantitatively evaluated for histopathological damage to the structure of bronchioles, alveoli and pulmonary blood vessels. Scores were given to compare the severity of lung damage between different mice. A score of 0-3 was given for each category of damage, and the highest total score for each sample was calculated (highest possible total score = 9). Scale bar: 200 µm. Images are representative of three independent experiments. Error bars represent mean $\pm$ SD. Statistical analysis was performed using one-way ANOVA followed by Dunn's multiple comparisons test: *** $p < 0.001$, ** $p < 0.01$, ns: not significant. Hamster cartoon created with BioRender.com.

Supplementary Fig.S6

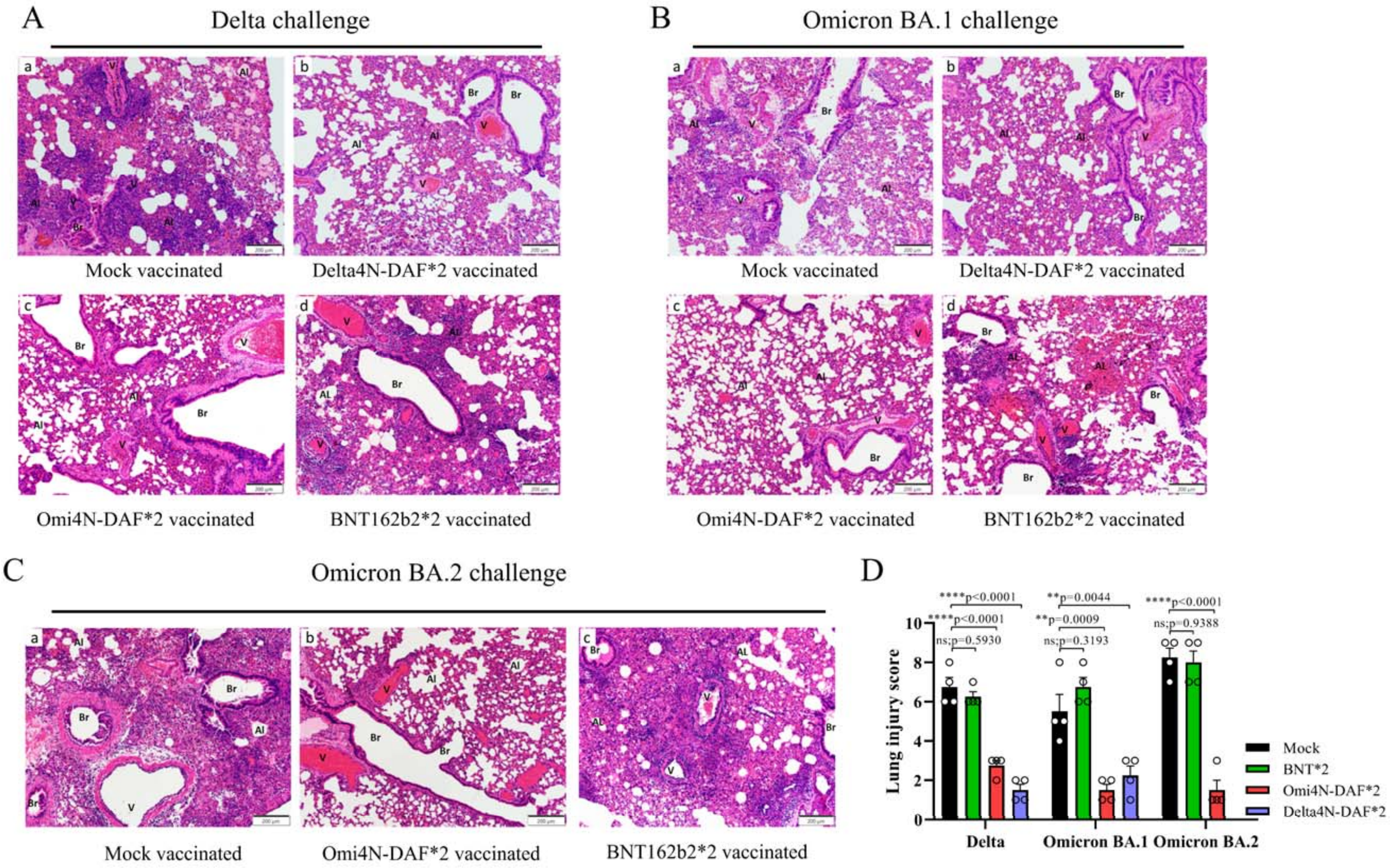

Fig. S6 Histopathological analysis of lung pathology in immunized-challenged hamsters.

Hamsters were prime-boost vaccinated intranasally with Delta4N-DAF (2 x 10^6 pfu), Omi4N-DAF (2 x 10^6 pfu) or PBS (Mock) or intramuscularly with BNT162b2 mRNA (n=4 for each group). Four weeks after boost immunization, hamsters were challenged with 1 x 10^4 pfu of the SARS-CoV-2 variants Delta, Omicron BA.1 or Omicron BA.2 (n=4 for each group). Lungs were collected, fixed, processed into paraffin blocks, and sections H&E stained at 4 dpi. (A) Mock-vaccinated or immunized hamsters challenged with Delta virus. a. The image of the lung at day 4 post infection showed diffuse histopathological changes, including a larger area of alveolar consolidation with immune cell infiltration and alveolar space exudation. A bronchial section (Br) showed epithelium detached in the lumen; three pulmonary blood vessel sections (V) showed severe perivascular and endothelial immune cell infiltration. b. The lung section showed two bronchial sections (Br) with no obvious epithelial damage or immune cell infiltration; the blood vessels (V) showed no indication of inflammatory infiltration. The alveolar wall (Al) showed blood vessel congestion, but no consolidation. c. A mild degree of peribranchial infiltration (Br) and alveolar septum infiltration (Al) was observed, while blood vessels appeared normal. d. The image shows a larger area of alveolar (Al) consolidation with immune cell infiltration and alveolar space exudation. A bronchial section (Br) showed a mild degree of luminal cell debris. The two pulmonary blood vessel sections (V) showed perivascular and mild to moderate endothelial immune cell infiltration. (B) Mock-vaccinated or immunized hamsters challenged with Omicron BA.1 virus. a. A larger area of alveolar (Al) immune cell infiltration and alveolar space exudation. A bronchial section (Br) displayed peribronchiolar infiltration and luminal cell debris. Two sections of pulmonary blood vessels (V) showed mild inflammatory changes and vessel wall infiltration. b. The lung section showed bronchial infiltration and luminal cell debris (Br); the blood vessel (V) showed no appearance of inflammatory infiltration. The alveolar wall (Al) showed blood vessel congestion, but no alveolar space infiltration or exudation. c. The lung section appeared largely normal. d. The Omicron BA.1 challenged lung showed vessel congestion and a patchy area of alveolar (Al) hemorrhage and immune cell infiltration, together with a mild degree of bronchial luminal cell debris (Br) and perivascular immune cell infiltration (V). (C) Mock-vaccinated or immunized hamsters challenged with Omicron BA.2 virus. a. A larger area of alveolar (Al) consolidation due to immune cell infiltration and alveolar space exudation. Bronchial epithelium desquamation with luminal cell debris is shown in the three sections (Br). A large blood vessel (V) showed perivascular edema, infiltration and a moderate degree of infiltration. b. Beside congestion of the large blood vessel (V), the alveolar and bronchial structures are largely normal. c. Diffuse alveolar (Al) consolidation. Severe perivascular and endothelial infiltration (V) were observed in the two pulmonary blood vessels. Though the bronchial lumen is filled with hemorrhagic secretions, epithelial damage is less obvious. Br, bronchiole; V, blood vessel, Al, alveolar structure. Scale bar: 200 µm. Images are representative of three independent experiments. (D) The lung sections were quantitatively evaluated for histopathological damage to the structure of bronchioles, alveoli and pulmonary blood vessels. Scores were given to compare the severity lung damages in different hamsters. A score of 0-3 was given to each category of damage, and the highest total score for each sample was calculated (highest possible total score = 9). Scale bar: 200 µm. Images are representative of three independent experiments. Error bars represent mean $\pm$ SD. Statistical analysis was performed using one-way ANOVA followed by Dunn's multiple comparisons test: **** $p < 0.0001$, ** $p < 0.01$, ns: not significant.

Supplementary Fig.S7

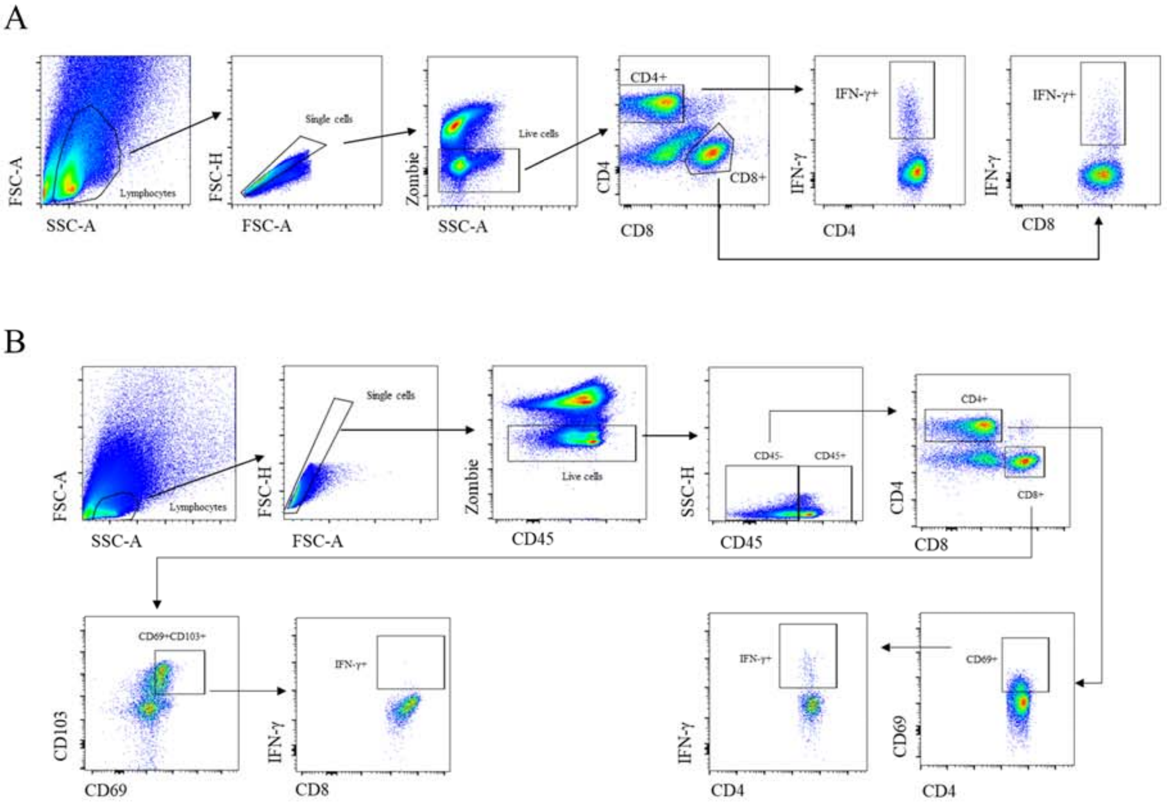

Fig. S7 Flow cytometry gating strategy.

The cells were stimulated by a SARS-CoV-2 RBD or influenza-NP peptide pool (Supplementary Table 2), stained for cell surface markers and intracellular cytokines and flow cytometry performed with gating with FSC-A vs SSC-A to exclude debris, then FSC-H vs FSC-A to select single cells, and then gating with Zombie vs SSC-A or CD45 for live cells. (A) Gating strategies to identify acute phase IFN-γ+ CD4+ T cells and IFN-γ+ CD8+ T cells in lungs. (B) Gating strategies to identify memory phase CD45- IFN-γ+ CD69+ CD4+ T cells and CD45- IFN-γ+ CD69+ CD103+ CD8+ T cells in lungs 5 weeks after immunization.

Supplementary Table 1

Table S1. Mouse adaptations present in plaque purified SARS-CoV-2 Gamma-MA and Omicron-MA relative to the parental SARS-CoV-2 strain. NSP, nonstructural protein.

Gamma-MA			Omicron-MA		
Mutation	Gene	Coding Change	Mutation	Gene	Coding Change
A10458G	NSP5	D153G	A9489G	NSP4	H313R
C12060T	NSP8	S8F	T10931C	NSP5	F294L
T21706A	Spike	H66Q	G19468A	NSP14	G481S
A22743C	Spike	K417T	C23913T	Spike	T791I
			C28289T	N	L13F

Supplementary Table 1

Table S2. List detailing the SARS-CoV-2 RBD peptide pool (15-mers overlapping by 11 residues, spanning the RBD sequence of Spike (331-531)) used in this study.

Peptide		Delta4N	Omi4N	Peptide		Delta4N	Omi4N
1	NLCPFGEVFNATRFA			24	PDD*FTGCVIAWNSNN*	D428N	D428N,N440K
2	FGEVFNATRFASVYA			25	TGCVIAWNSNN*LDSK		N440K
3	FNATRFASVYAWNRK			26	IAWNSNN*LDSKVG*GN		N440K,G446S
4	RFASVYAWNRKRISN			27	SNN*LDSKVG*GNYNYL*	L452R	N440K,G446S
5	VYAWNRKRISNCVAD			28	DSKVG*GNYNYL*YRLF	L452R	G446S
6	NRKRISNCVADYSVL			29	G*GNYNYL*YRLFRKSN	L452R	G446S
7	ISNCVADYSVLYNSA*	A372T	A372T	30	NYL*YRLFRKSNLKPF	L452R	
8	VADYSVLYNSA*SFST	A372T	A372T	31	RLFRKSNLKPFERDI		
9	SVLYNSA*SFSTFKCY	A372T	A372T	32	KSNLKPFERDISTEI		
10	NSASFSTFKCYGVSP			33	KPFERDISTEIYQAG		
11	FSTFKCYGVSPTKLN			34	RDISTEIYQAGS*T*PC	T478K	S477N, T478K
12	KCYGVSPTKLNDLCF			35	TEIYQAGS*T*PCNGVE*	T478K	S477N, T478K, E484A
13	VSPTKLNDLCFTNVY			36	QAGS*T*PCNGVE*GFNC	T478K	S477N, T478K, E484A
14	KLNDLCFTNVYADSF			37	T*PCNGVE*GFNCYFPL	T478K	T478K, E484A
15	LCFTNVYADSFVIRG			38	GVE*GFNCYFPLQ*SYG*		E484A, Q493R, G496S
16	NVYADSFVIRGDEVR			39	FNCYFPLQ*SYG*FQ*PT		Q493R, G496S, Q498R
17	DSFVIRGDEVRQIAP			40	FPLQ*SYG*FQ*PTN*GVG		Q493R, G496S, Q498R, N501Y
18	IRGDEVRQIAPG*QTG	G413N	G413N	41	SYG*FQ*PTN*GVGY*QPY		G496S, Q498R, N501Y, Y505H
19	EVRQIAPG*QTGKIAD	G413N	G413N	42	Q*PTN*GVGY*QPYRVVV		Q498R, N501Y, Y505H
20	IAPG*QTGKIADYNYK	G413N	G413N	43	GVGY*QPYRVVVLSFE		Y505H
21	QTGKIADYNYKLPDD*	D428N	D428N	44	QPYRVVVLSFELLHA		
22	IADYNYKLPDD*FTGC	D428N	D428N	45	VVVLSFELLHAP*ATV	P521N	P521N
23	NYKLPDD*FTGCVIAW	D428N	D428N	46	SFELLHAP*ATVCGPK	P521N	P521N

* Variations in Delta4N and Omi4N from the sequences of the peptide pool are indicated in the table.
