# Supplementary material for: An intranasal influenza virus-vectored vaccine prevents SARS-CoV-2 replication in respiratory tissues of mice and hamsters
Source: Nat Commun. 2023 Apr 12;14:2081. doi: 10.1038/s41467-023-37697-1 (PMC10092940; doi:10.1038/s41467-023-37697-1)
Supplement: Supplementary file 2 — Reporting Summary [file 41467_2023_37697_MOESM2_ESM.pdf]

nature portfolio

Corresponding author(s): P Wang and H Chen

Last updated by author(s): Mar 3, 2023

Reporting Summary

Nature Portfolio wishes to improve the reproducibility of the work that we publish. This form provides structure for consistency and transparency in reporting. For further information on Nature Portfolio policies, see our Editorial Policies and the Editorial Policy Checklist.

Statistics

For all statistical analyses, confirm that the following items are present in the figure legend, table legend, main text, or Methods section.

n/a	Confirmed	
☐	☒	The exact sample size (*n*) for each experimental group/condition, given as a discrete number and unit of measurement
☐	☒	A statement on whether measurements were taken from distinct samples or whether the same sample was measured repeatedly
☐	☒	The statistical test(s) used AND whether they are one- or two-sided<br>*Only common tests should be described solely by name; describe more complex techniques in the Methods section.*
☒	☐	A description of all covariates tested
☒	☐	A description of any assumptions or corrections, such as tests of normality and adjustment for multiple comparisons
☐	☒	A full description of the statistical parameters including central tendency (e.g. means) or other basic estimates (e.g. regression coefficient) AND variation (e.g. standard deviation) or associated estimates of uncertainty (e.g. confidence intervals)
☐	☒	For null hypothesis testing, the test statistic (e.g. *F*, *t*, *r*) with confidence intervals, effect sizes, degrees of freedom and *P* value noted<br>*Give P values as exact values whenever suitable.*
☒	☐	For Bayesian analysis, information on the choice of priors and Markov chain Monte Carlo settings
☒	☐	For hierarchical and complex designs, identification of the appropriate level for tests and full reporting of outcomes
☒	☐	Estimates of effect sizes (e.g. Cohen's *d*, Pearson's *r*), indicating how they were calculated

Our web collection on statistics for biologists contains articles on many of the points above.

Software and code

Policy information about availability of computer code

Data collection	Confocal images were captured by LSM 700 (Carl Zesis).<br>Flow cytometry data was done by BD FACSAria III cell sorter (BD).<br>Luminance data was acquired by Victor III micropate reader (PE).<br>Western blot data was acquired by Odyssey scanner (LICOR).
Data analysis	Statistical analysis was done Graphpad prism Verison 8.<br>Flow cytometry data was analyzed by FlowJo V9.

For manuscripts utilizing custom algorithms or software that are central to the research but not yet described in published literature, software must be made available to editors and reviewers. We strongly encourage code deposition in a community repository (e.g. GitHub). See the Nature Portfolio guidelines for submitting code & software for further information.

Data

Policy information about availability of data

All manuscripts must include a data availability statement. This statement should provide the following information, where applicable:

- Accession codes, unique identifiers, or web links for publicly available datasets
- A description of any restrictions on data availability
- For clinical datasets or third party data, please ensure that the statement adheres to our policy

The data of this study are available upon reasonable request.

Human research participants

Policy information about studies involving human research participants and Sex and Gender in Research.

Reporting on sex and gender	*Use the terms sex (biological attribute) and gender (shaped by social and cultural circumstances) carefully in order to avoid confusing both terms. Indicate if findings apply to only one sex or gender; describe whether sex and gender were considered in study design whether sex and/or gender was determined based on self-reporting or assigned and methods used. Provide in the source data disaggregated sex and gender data where this information has been collected, and consent has been obtained for sharing of individual-level data; provide overall numbers in this Reporting Summary. Please state if this information has not been collected. Report sex- and gender-based analyses where performed, justify reasons for lack of sex- and gender-based analysis.*
Population characteristics	*Describe the covariate-relevant population characteristics of the human research participants (e.g. age, genotypic information, past and current diagnosis and treatment categories). If you filled out the behavioural & social sciences study design questions and have nothing to add here, write "See above."*
Recruitment	*Describe how participants were recruited. Outline any potential self-selection bias or other biases that may be present and how these are likely to impact results.*
Ethics oversight	*Identify the organization(s) that approved the study protocol.*

Note that full information on the approval of the study protocol must also be provided in the manuscript.

Field-specific reporting

Please select the one below that is the best fit for your research. If you are not sure, read the appropriate sections before making your selection.

☒ Life sciences ☐ Behavioural & social sciences ☐ Ecological, evolutionary & environmental sciences

For a reference copy of the document with all sections, see nature.com/documents/nr-reporting-summary-flat.pdf

Life sciences study design

All studies must disclose on these points even when the disclosure is negative.

Sample size	All experiments were repeated at least three times to give a n number of 3 or above. A n number equals to 3 is the the standard of biological experiments. No sample size calculation was performed. Sample size is chosen based on the standard of the corresponding field (PMID: 32215622).
Data exclusions	No data was excluded.
Replication	All experiments were repeated at least once. Similar findings were obtained from all repeats.
Randomization	Randomization was applied to the grouping of the animals. Animals were randomly allocated to the groups.
Blinding	No blinding was done. Blinding was not relevant to the study because the results are quantitative and objective, and does not require a subjective judgment.

Reporting for specific materials, systems and methods

We require information from authors about some types of materials, experimental systems and methods used in many studies. Here, indicate whether each material, system or method listed is relevant to your study. If you are not sure if a list item applies to your research, read the appropriate section before selecting a response.

Materials & experimental systems

n/a	Involved in the study	
[ ]	[x]	Antibodies
[ ]	[x]	Eukaryotic cell lines
[x]	[ ]	Palaeontology and archaeology
[ ]	[x]	Animals and other organisms
[x]	[ ]	Clinical data
[x]	[ ]	Dual use research of concern

Methods

n/a	Involved in the study	
[x]	[ ]	ChIP-seq
[ ]	[x]	Flow cytometry
[x]	[ ]	MRI-based neuroimaging

Antibodies

Antibodies used

In house rabbit anti-SARS-CoV-2 Spike RBD antibody, 1:1000 for western blot.
In house mouse anti-SARS-CoV-2 Spike RBD antibody, 1:100 for immunofluorescence assay.
In house mouse anti-influenza nucleoprotein antibody, 1:5000 for western blot.
Rat Anti-mouse CD8a-PerCP-Cy5 (Biolegend #100734), 1: 200 for flow cytometry staining.
Rat Anti-mouse CD4-V450 (BD #560468),1: 200 for flow cytometry staining.
Rat Anti-mouse IFNg-APC(Biolegend #505810), 1: 100 for flow cytometry staining.
Rat Anti-mouse TNFa-PE (Biolegend #506306), 1: 100 for flow cytometry staining.
Rat Anti-Mouse CD45-PerCP-Cy5.5(BD #550994), 1:100 for flow cytometry staining.
Rat Anti-Mouse CD69-BV711(Biolegend #104537), 1:100 for flow cytometry staining.
Rat Anti-Mouse CD103-BV421(Biolegend #121422), 1:100 for flow cytometry staining.
Zombie Aqua™ Fixable Viability Kit (Biolegend #423102), 1:200 for flow cytometry staining.
Rat Anti-Mouse CD4-FITC(Biolegend #100406), 1:100 for flow cytometry staining.
Rat Anti-Mouse CD8-APC/Fire 750(Biolegend #100766), 1:100 for flow cytometry staining.

Validation

The commercial antibodies were validated by the manufacturers.
The in-house anti-SARS-CoV-RBD immune serum were validated with ELISA, Western blots, and immunofluorescence staining in the previous publication (PMID: 32835326).
The in-house anti-influenza nucleoprotein antibody were validated Western blots and immunofluorescence staining in the previous publication (PMID: 25043584).

Eukaryotic cell lines

Policy information about cell lines and Sex and Gender in Research

Cell line source(s)

MDCK, A549, BHK21, 293T and VERO-E6 was obtained from ATCC.
In house 293T-hACE2 cell line was prepared by lentivral transduction of 293T cells.
VEROE6-TMPRSS2 was a gift from Dr. Kelvin To at the department of microbiology, HKU.

Authentication

ATCC cell lines were authenticated by ATCC.
The expression of ACE2 in 293T-hACE2 was verified by qRT-PCR.

Mycoplasma contamination

All cell lines were tested negative by PCR for mycoplasma.

Commonly misidentified lines
(See ICLAC register)

Nil

Animals and other research organisms

Policy information about studies involving animals; ARRIVE guidelines recommended for reporting animal research, and Sex and Gender in Research

Laboratory animals

6-8 weeks old male Golden Syrian hamster and 6-8 weeks old BALB/c mice were used in this study.

Wild animals

Nil

Reporting on sex

Golden Syrian hamster: Male, BALB/c mice: female

Field-collected samples

Nil

Ethics oversight

All animal studies were approved by the Committee on the Use of live animals in Teaching and Research at HKU.

Note that full information on the approval of the study protocol must also be provided in the manuscript.

Flow Cytometry

Plots

Confirm that:

☒ The axis labels state the marker and fluorochrome used (e.g. CD4-FITC).

☒ The axis scales are clearly visible. Include numbers along axes only for bottom left plot of group (a 'group' is an analysis of identical markers).

☒ All plots are contour plots with outliers or pseudocolor plots.

☒ A numerical value for number of cells or percentage (with statistics) is provided.

Methodology

Sample preparation	Splenocytes were isolated through cell strainer (BD) and resuspended in RPMI medium (10% FBS and P/S). Lung tissues were chopped and digested with collagenase (Sigma) and DNAse (Roche) for 1h at 37 0C. Red blood cells were lysed by Lysing solution (BD). After washes, cells were counted and resuspended in RPMI solution.
Instrument	BD FACSAria III cell sorter
Software	FlowJo V9
Cell population abundance	No sorting was done.
Gating strategy	Cells were gated with different strategies:<br>1. FSC-H vs FSC-A for single cells, Zombie dye VS SSC-A for live cells, CD4 vs CD8 for T cell subsets, different cytokines VS CD4 or CD8.<br>2. FSC-H vs FSC-A for single cells, Zombie dye VS SSC-A for live cells, CD45 for circulating cells or Lung tissue resident cells, CD4 vs CD8 for T cell subsets, CD69 and CD103 for tissue resident markers, different cytokines VS CD4 or CD8.

☒ Tick this box to confirm that a figure exemplifying the gating strategy is provided in the Supplementary Information.
